# 6.F. Skills building seminar: Digital health and communication

**DOI:** 10.1093/eurpub/ckac129.361

**Published:** 2022-10-25

**Authors:** Organised by: EUPHA-DH, Chair persons: Stefan Buttigieg (EUPHA-DH), Anthony Staines (Ireland)

## Abstract

Information has always been central to public health practice. As digital health develops it becomes increasingly central to health care delivery, health system management, and population health monitoring. Despite enormous investment, and great effort in collecting these data, much of the potential value of health care information is lost because of poor data architecture. Good design and interoperability are sometimes seen as obstacles to implementation. but in fact they are key to getting value out of health data systems. Tools and ideas developed over the last thirty years by bioinformaticians may be quite unfamiliar to public health practitioners. The intention of this seminar is to introduce some of these ideas, specifically syntactic and semantic interoperability, ontologies, and terminologies, and to show, using case studies, how these may fit into public health practice.

**Key messages:**

• It is necessary to have an excellent data architecture to get a good return on investment in healthcare ICT systems.

• Standards are key to interoperability, be that syntactic or semantic.

